# The importance of registries in clinical practice: Insights from the national atopic dermatitis registry TREATgermany 

**DOI:** 10.5414/ALX02545E

**Published:** 2025-03-06

**Authors:** Tatjana Honstein, Barbara Kind, Dora Stölzl, Inken Harder, Sigrid Müller, Thomas Birkner, Luise Heinrich, Sascha Fischer, Nicole Sander, Annice Heratizadeh, Jochen Schmitt, Stephan Weidinger, Thomas Werfel

**Affiliations:** 1 Division of Immunodermatology and Allergy Research, Department of Dermatology and Allergy, Hannover Medical School, Hannover,,; 2 Center for Evidence-Based Healthcare, University Hospital Carl Gustav Carus and Carl Gustav Carus Faculty of Medicine, Technische Universität Dresden, Dresden, and; 3 Department of Dermatology, Venereology and Allergology, Schleswig-Holstein University Hospital, Kiel Campus, Kiel, Germany; *Shared first authorship

**Keywords:** atopic dermatitis, TREATgermany, registry, public health research, systemic treatment, biomarkers

## Abstract

Atopic dermatitis (AD) is an inflammatory, multifactorial skin disease characterized by eczematous skin lesions, severe itching, and serious limitations in quality of life. Since 2016, TREATgermany has been a national clinical registry for patients with moderate to severe AD with around 2,550 participating patients, enabling the collection of physical, patient-reported social and psychological data as well as the collection of biosamples in routine care. TREATgermany is one of the largest academically managed AD registries in the world. This current review summarizes a selection of published analyses from registry data from TREATgermany, which retrospectively identified important correlations with regard to treatment response and safety, and investigated quality of life, performance, and mental health under everyday conditions. In addition, initial molecular signatures were identified from the blood and skin samples obtained from the patients included in the registry, which may be useful as prognostic markers.

## Introduction 

Atopic dermatitis (AD) is a chronic inflammatory skin condition characterized by intense itching, eczematous skin lesions, and a chronically relapsing disease progression. It affects up to 20% of children and 10% of adults [1, 2]. Those affected experience significant personal impacts on their quality of life. The burden on the healthcare system caused by the disease is considerable [2, 3, 4]. Due to the high direct and indirect costs, AD is regarded as a particularly burdensome skin disease, with an estimated economic impact of over 2.2 billion euros and average out-of-pocket expenses of 941 euros per year [5, 6, 7, 8, 9, 10]. 

TREATgermany is the national clinical registry for patients with moderate to severe AD in Germany. Established in 2016, the registry has been managed since January 2020 by a consortium of four partners, the German Society for Allergology and Clinical Immunology (DGAKI) WissenschaftsGmbH, the Center for Evidence-based Healthcare (ZEGV) of the Faculty of Medicine at the Technical University of Dresden (Prof. Jochen Schmitt), the coordination centers at the Hannover Medical School (Prof. Thomas Werfel), and the University Hospital Schleswig-Holstein in Kiel (Prof. Stephan Weidinger). Furthermore, the registry is supported by the DGAKI and the German Dermatological Society (DDG) as scientific sponsors as well as financially by pharmaceutical companies and is part of a network with national AD registries throughout Europe (TREATeurope, Dream to TREAT AD). It is also a model registry for “target trial emulation” in a project funded by the Federal Ministry of Health. In addition to physician-reported parameters, the results of patient reports are also documented in a structured and prospective manner as part of routine medical care. In the optional additional bioanalytics module, various biosamples are obtained which form a valuable database for molecular analyses and the investigation of biomedical correlations. 

The main objective of the registry is to gain insights into care patterns in patients with moderate to severe AD in real-life healthcare settings and to investigate the efficacy, tolerability, and safety of available therapies with a particular focus on the patient perspective. [Table TableAbb]

## Results 

### Current status of the TREATgermany registry 

**Figure 1.**
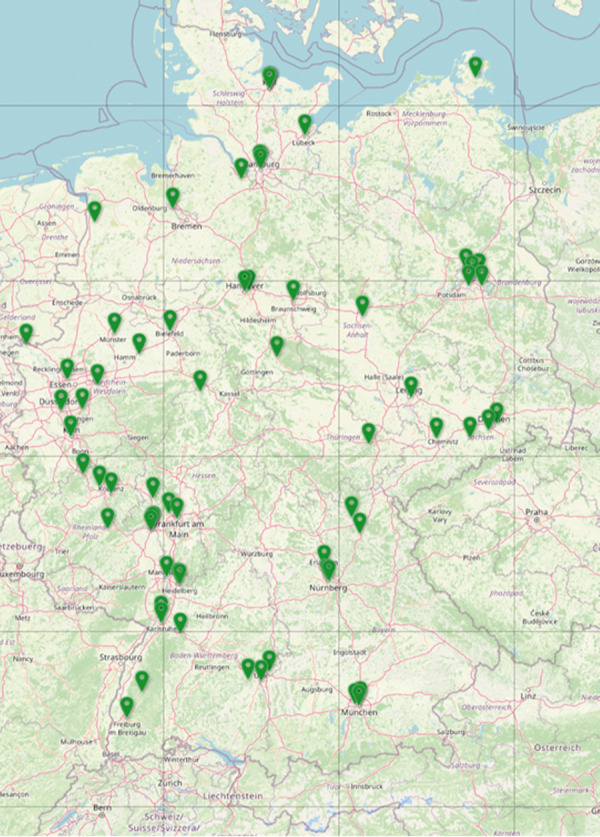
Participating centers of TREATgermany registry.

By August 2024, 2,250 adult patients from all over Germany are included in TREATgermany. Data is currently being collected multicentrically in 62 dermatology practices and 28 clinics. Biosamples for molecular analysis are also collected in 21 of these centers (Figure 1). Based on the constantly growing data set, it has so far been possible to gain insights into socio-demographic characteristics, system therapies, and molecular signatures (Table 1). 

### Drug survival, effectiveness, safety, and implementation of system therapies in routine care 

In 2020, the first data on the implementation of dupilumab in routine care was published from the registry. 137 patients who received systemic therapy with dupilumab at the time and were included in the registry had a high disease burden at baseline with mean Eczema Area Severity Index (EASI) and Objective Scoring Atopic Dermatitis (oSCORAD) values of 22.9 ± 13.6 and 48.0 ± 15.7, respectively. Three months after the start of treatment, a significant reduction in severity was measured in 105 patients who were evaluable at the time (mean EASI: 6.1 ± 6.1, p < 0.001; mean oSCORAD: 22.3 ± 11.4, p < 0.001). The response rates for EASI50 (i.e., a 50% reduction in the eczema score EASI), for EASI75, and for EASI90 were 77.1, 57.1, and 25.7%, respectively, and were slightly higher on average than when considering the eczema score oSCORAD (EASI mean percentage change: 75.2%; oSCORAD: mean percentage change: 54.7% after 3 months). The patient-reported values relating to quality of life, sleep, and itching also improved significantly after 3 months (Patient-Oriented Eczema Measure (POEM) 19.3 ± 6.4 vs. 8.8 ± 5.9, p < 0.001; Dermatological Life Quality Index (DLQI) 12.4 ± 6.7 vs. 4.4 ± 5.2, p < 0.001; numerical rating scale (NRS) pruritus/sleeping 6.4 ± 2.2 vs. 2.7 ± 2.1, p < 0.001 and 5.4 ± 3.0 vs. 1.5 ± 2.1, p < 0.001). Within the first 3 months, 13.3% of patients developed conjunctivitis, after 6 months the figure was 23%, which was comparable to the results from phase III studies [11]. 

An extended analysis compared dupilumab with cyclosporin A (CyA) under real-life care conditions. Both drugs showed similar efficacy with comparable baseline characteristics (3 months after the start of therapy dupilumab vs. CyA: EASI50 75.4 vs. 80.5%; EASI75 48.7 vs. 58.5%; EASI90 22.9 vs. 31.7%), but the discontinuation rates for CyA were significantly higher. Dupilumab was discontinued after 12 and 24 months in 5.0 and 11.4% of cases, respectively, while 78 and 100% of CyA patients discontinued treatment. The main reasons for discontinuation of CyA were side effects (31%) and insufficient efficacy (27%); furthermore, treatment was possibly discontinued in a subgroup of patients in line with the guidelines, also because the duration of treatment with CyA was already reached [12]. 

In 2024, the first registry data from TREATgermany on patients treated with the JAK inhibitor baricitinib in routine care were published [13]. A defined cohort of patients who initially received baricitinib without any systemic therapy change and who attended follow-up visits under this therapy showed a good response. Significant improvements in oSCORAD (45.9 ± 12.3 vs. 28.2 ± 15.5, p < 0.001) and EASI (21.5 ± 13.2 vs. 9.3 ± 9.0, p < 0.001) were measured after 3 months. The response rates for EASI50 and EASI75 were 60.0 and 44.0%, respectively. In addition, quality of life (DLQI: 15.2 ± 7.5 vs. 6.2 ± 6.4) and AD severity from the patient’s perspective (POEM: 20.2 ± 6.3 vs. 9.3 ± 6.9) improved significantly [13]. 

### Itching as the key symptom in patients with moderate to severe AD 

In two analyses from 2023, itching was examined within the registry. In both studies, patient-reported itch scores were used, determined using an NRS (scale: 0 – 10) and documented for the last 3 days prior to the initial examination. The results of the first analysis showed a moderate correlation between itch intensity and physician-rated AD severity: oSCORAD (r = 0.44 (0.39 – 0.48)), EASI (r = 0.41 (0.36 – 0.46)), and Investigator Global Assessment (IGA) (r = 0.46 (0.42 – 0.51)). A strong correlation was found between itching and patient-reported disease intensity, severity and quality of life as measured by the Patient Global Assessment (PGA) (r = 0.68 (0.65 – 0.71)), POEM (r = 0.66 (0.63 – 0.69)), and the DLQI (r = 0.61 (0.57 – 0.65)). In addition, the relationship between AD-related sleep loss within the last 3 days and itching was investigated. A strong correlation was found here (r = 0.75 (0.72 – 0.78)) [14]. 

In the second analysis, the correlations between itching and topical as well as systemic therapy were examined in patients who entered the registry between June 2016 and April 2021. Of 1,061 patients with topical therapy, 89.9% received glucocorticoids (TCS), 39.7% tacrolimus, 33.6% pimecrolimus, and 35.7% UV phototherapy. When examining itch intensity and topical treatments in the last 12 months, patients using tacrolimus (users: 5.5 ± 2-7, n = 447; non-users: 5.9 ± 2.7; n = 674; p = 0.008) and pimecrolimus (users: 5.5 ± 2.6, n = 373; non-users: 5.8 ± 2.8, n = 748; p = 0.047) showed significantly lower itch scores. Among patients on systemic treatment (31.2%), dupilumab patients had the lowest itch scores (4.0 ± 2.8), while CyA patients reported mean scores of 4.9 ± 2.8 and systemic glucocorticosteroids patients reported mean scores of 6.3 ± 2.8 for itch on a scale of 0 – 10 [15]. 

### Moderate to severe AD leads to negative impact on health economy 

An analysis performed in 2018 examined the link between disease-related quality of life, as measured by the DLQI, and occupational performance limitations, recorded with the Work Limitations Questionnaire (WLQ) in adult patients in the TREATgermany registry. Employed patients with moderate to severe AD showed an average loss of productivity of almost 10%. Around 9.7% of registry patients also reported to suffer from diagnosed depression. Compared to patients without depression, these patients were significantly older (45.2 ± 13.3 years vs. 41.5 ± 14.3 years, p = 0.021), reported significantly more impairment in quality of life (DLQI 14.6 ± 8.3 vs. 11.4 ± 7.7, p < 0.001), and achieved a higher Center for Epidemiologic Studies Depression Score (CES-D; 23.9 ± 12.0 vs. 13.4 ± 9.1, p < 0.001) [16]. 

The analysis of data from 228 employed registry patients revealed a 6% loss of productivity compared to a healthy comparison group. Here, the WLQ showed moderate associations with itching (NRS itching/last 3 days, r = 0.3) and sleep loss (NRS sleep/last 3 days, r = 0.4) as well as strong correlations with depressive symptoms (CES-D score, r = 0.7) and fatigue (mean Fatigue Severity Scale (FSS) score, r = 0.6). Patients reported limitations in time management (25.7%), in cognitive-social activities (20.0%), and in physical work (20.3%) [17]. 

### Eczema herpeticum in patients with moderate to severe AD 

The prevalence and recurrence rate of eczema herpeticum (EH) was investigated in 893 patients from the TREATgermany registry. 21.8% (n = 195) of the registry patients reported at the baseline visit that they had had at least 1 EH event. 54.9% of these EH registry patients documented having experienced multiple episodes of the disease. Patients with EH were older than patients without a history of EH. To assess the risk of atopic comorbidities for EH, physician- and patient-reported comorbidities were analyzed. Patients with EH were more likely to have asthma (59.8 vs. 50.8%, p = 0.03) and rhinitis (76.9 vs. 68.9%, p = 0.03). Although total IgE was not a suitable marker to identify EH patients, specific sensitization to house dust mites (80.0 vs. 65.9%, p < 0.01), mold (49.3 vs. 32.4%, p < 0.01), and food (58.2 vs. 43.2%, p < 0.01) was significantly more common in patients with a history of EH compared to those who had not experienced EH [18]. 

### Molecular genetic indications of disease progression and therapy response 

Lesional and non-lesional skin biopsies from 57 registry patients before and 12 weeks after initiation of treatment with dupilumab (n = 22) or CyA (n = 8) and skin biopsies from 31 healthy controls were subjected to mRNA sequencing. Both lesional and non-lesional skin samples from AD patients showed a stable “core” signature with impaired epidermal differentiation and activation of IL-31/IL-1 signaling pathways compared to skin samples from healthy controls. When comparing lesional to non-lesional skin, a “dynamic signature” of lesions was observed with enrichment of transcripts for molecules of type 2 inflammation, Th17 signaling, and natural killer (NK) cell molecules. 

The target of dupilumab, IL-4Rα, was one of three major dysregulated genes in lesional skin. Systemic treatments with dupilumab or CyA significantly reduced the mRNA expression of type-2-associated molecules, but the residual profile remained fundamentally different from healthy skin [19]. The skin transcriptome was then analyzed with a focus on NK cell signatures in 57 patients and 31 healthy controls. After 12 weeks of systemic treatment (dupilumab n = 21, CyA n = 8), lesional skin still showed a strong upregulation of NK cell markers that was not completely abrogated by treatment. Additional immunofluorescence staining showed an increased number of NK cells in lesional compared to non-lesional and healthy skin. Lesional AD skin exhibits NK cell dysregulation that is only partially reversible despite therapy and may represent an underestimated disease mechanism [20]. 

A transcriptome analysis published in 2022 even enabled a first classification into endotypes. The blood transcriptome of AD patients at baseline showed clear inflammatory expression patterns that could be divided into two clusters: eosinophil-high and eosinophil-low. The eosinophil-high profile showed a stronger global dysregulation and a positive correlation between disease activity and signatures related to IL-5 signaling. In contrast, the eosinophil-low profile showed low transcriptional dysregulation and no association between disease activity and gene expression. Interestingly, clinical improvement in AD with dupilumab was associated with a decrease in innate immune response and an increase in lymphocyte signatures, including B-cell activation and NK-cell composition/function [21]. Initial results with indications of certain endotypes could be a further step towards precision medicine [21]. 

Analyses from the registry also showed that new treatments such as dupilumab and baricitinib have a similar safety and efficacy profile in clinical practice as in the clinical trials [12, 13]. 

## Discussion and summary 

AD is heterogeneous and multifactorial, therefore many aspects are still insufficiently characterized. The search for specific biomarkers for AD with the aim of taking further steps towards precision medicine is not yet complete. With the growing variety of treatment options, it is becoming more and more conceptually attractive to select the “right” treatment for a patient based on clinical characteristics and biomarkers. The growing “biobank” with blood and skin samples from the TREATgermany register can certainly contribute new findings in the future. 

Besides the physician’s assessment, the patients’ goals with regard to their disease and treatment are increasingly becoming the focus of shared decision making. This includes factors that affect disease progression, other health conditions, and the patient’s quality of life [16, 22]. In this area, findings from real-life scenarios such as the TREATgermany register can contribute new insights. 

Socio-demographic aspects are crucial for identifying risk factors, understanding the prevalence and severity of the disease, and developing suitable treatment approaches. With regard to new therapies, the registry ensures a very short response time through continuous, prospective recruitment in order to consider the implementation of new therapies in clinical routine and to identify safety, efficacy and, if necessary, molecular biomarkers [11, 12, 23]. 

Clinical registries such as TREATgermany are essential to generate complementary information on clinical trials to support evidence-based decisions in routine care. They also make it possible to investigate the integration of medical innovations, such as new therapies, into practice and their effectiveness under real-life conditions, which can be helpful for the evaluation of treatment strategies. 

## Acknowledgement 

The authors would like to thank the participating patients, doctors, and medical staff, the documentation team and, not least, the TREATgermany study group listed at treatgermany.org for their essential contributions to this work. 

## Authors’ contributions 

TH, BK, DS, IH performed the literature search, drafted and wrote the manuscript. 

TH, BK, DS, IH and AH revised the manuscript. AH, JS, SW, TW were responsible for the concept and revising of the manuscript. All authors reviewed and approved the final version of the manuscript and its submission. 

## Funding 

TREATgermany is an academic, investigator-initiated clinical disease registry that is ﬁnancially supported by AbbVie Deutschland GmbH & Co. KG, Almirall Hermal GmbH, Galderma, LEO Pharma GmbH, Lilly Deutschland GmbH, Pfizer, and Sanofi. 

## Conflict of interest 

SW has received institutional research grants from LEO, Pfizer, Sanofi and has served as a consultant and lecturer for AbbVie, Almirall, Eli Lilly, Galderma, Kymab, LEO Pharma, Pfizer, Sanofi, and Regeneron. JS has received institutional research grants from Novartis, Pfizer, GBA/Innovation Fund, BMG, BMBF, Free State of Saxony, Sanofi, ALK and has received honoraria for consulting activities. He is a member of the Expert Council on Health and Care at the BMG and the Government Commission. TW has received honoraria for lectures or scientific consulting from AbbVie, Almirall, Galderma, Janssen/JNJ, LEO Pharma, Leti, Lilly, Novartis, Pfizer, and Regeneron/Sanofi. AH has received honoraria for lectures or scientific consulting from Abbvie, Allmirall, ALK, LEO Pharma, Novartis, Pierre Fabre, Lilly, Sanofi, Genzyme, Ziarco, Beiersdorf, Hans Karrer, Klinge Pharma, Nutricia, and Meda. DS has received honoraria for lectures or scientific consulting from Sanofi, Regeneron, LEO Pharma, Abbvie, Almirall, and Novartis. TH received support for a research project from LEO Pharma. BK, IH, SM, TB, LH, SF, and NS have no conflict of interest to declare. 

**Abbreviations. TableAbb:** Abbreviations.

Atopic dermatitis	AD
Azathioprine	AZA
Center for Epidemiological Studies Depression Scale	CES-D
Center for Evidence-Based Healthcare	ZEGV
Cyclosporine A	CyA
Eczema Area Severity Index	EASI
Eczema herpeticatum	EH
Fatigue Severity Scale	FSS
German Society for Allergology and Clinical Immunology	DGAKI
German Society of Dermatology	DDG
Dermatological Life Quality Index	DLQI
Investigator Global Assessment	IGA
Janus kinase 1/2 inhibitor	JAK1/2-Inhibitor
Methotrexate	MTX
Mycophenolate-mofetil	MMF
Natural killer cells	NK-Cells
Objective Scoring Atopic Dermatitis	oSCORAD
Patient Global Assessment	PGA
Patient Oriented Eczema Measure	POEM
Topical Calcineurin Inhibitors	TCI
Topical Glucocorticoids	TCS
Work Limitations Questionnaire	WLQ

**Table 1.** Number of patients receiving systemic therapy in the TREATgermany registry from preliminary, unvalidated* data as of August 2024.

**Systemic therapy**	Baseline visit*	Follow-up visit**
Abrocitinib	66	57
Azathioprin	1	1
Baricitinib	67	63
Cyclosporin	96	83
Dupilumab	950	873
Glucocorticoide	52	33
Lebrikizumab	78	50
Methotrexate	8	7
Mycophenolate mofetil	2	2
Other	27	17
Tralokinumab	135	127
Upadacitinib	131	111
**Sum**	**1,613**	**1,424**

*The data validation will be performed with a time delay after monitoring; **in accordance with the study protocol, the first follow-up visit occurs 3 months after the baseline visit.
